# Impact of Abdominal Obesity on Thyroid Auto-Antibody Positivity: Abdominal Obesity Can Enhance the Risk of Thyroid Autoimmunity in Men

**DOI:** 10.1155/2020/6816198

**Published:** 2020-03-13

**Authors:** Jazyra Zynat, Suli Li, Yanrong Ma, Li Han, Fuhui Ma, Yuyuan Zhang, Bei Xing, Xinling Wang, Yanying Guo

**Affiliations:** Department of Endocrinology, People's Hospital of Xinjiang Uygur Autonomous Region, Ürümqi, Xinjiang, China

## Abstract

**Background:**

The interrelation between obesity and autoimmune thyroid diseases is complex and has not been confirmed. The aim of the present study was to observe the relationship between thyroid autoimmunity and obesity, especially abdominal obesity, in a large population.

**Methods:**

A total of 2253 residents who had lived in Xinjiang for more than 3 years were enrolled. Serum thyroid hormone concentration, thyroid autoantibodies, lipid parameters, Weight, height, and waist and hip circumference were measured.

**Results:**

The prevalence of thyroid peroxidase antibody (TPOAb) and/or thyroglobulin antibody (TgAb) positive was 32.1% (21.2% in men and 37% in women, *P* < 0.01). Compared with women, men had significantly higher TG levels, waist circumference, and hip circumference levels (*P* < 0.01), while women showed higher TSH, TPOAb, and TgAb levels (*P* < 0.01). The prevalence of overweight and obesity was 71.1% in men and 63.5% in women. Men had a higher prevalence of abdominal obesity than women (56.6% in men and 47.6% in women, *P* < 0.01). TPOAb correlates positively with waist circumference (*r* = 0.100, *P* < 0.05) in men. Binary logistic analysis showed that TPOAb positivity had increased risks of abdominal obesity in men, and the OR was 1.1044 (95% CI 1.035, 1.151, *P* < 0.05).

**Conclusion:**

Our results indicate that men had higher lipid levels, thicker waist circumference, and higher prevalence of overweight, obesity, and abdominal obesity. Abdominal obesity is a risk factor for TPOAb positivity in men, suggesting that abdominal obesity can enhance the risk of thyroid autoimmunity in men.

## 1. Introduction

Autoimmune thyroid diseases (AITDs) are the most common autoimmune diseases in humans; one of these is Hashimoto's thyroiditis (HT). HT is a common organ-specific autoimmune disorder, which presents the lymphocytic infiltration of the thyroid gland and the production of autoantibodies [1]. The major autoantigens in Hashimoto's disease are thyroid peroxidase (TPO) and thyroglobulin (Tg) antibodies, and the annual incidence of HT worldwide is estimated to be 0.3 to 1.5 cases per 1000 persons [2]. Intrathyroidal lymphocytic infiltration may lead to subclinical or overt hypothyroidism [3]; meanwhile, subclinical or overt hypothyroidism is an important risk factor for cardiovascular diseases [4]. Many studies reported that hypothyroidism causes a number of health issues, including insulin resistance, dyslipidemia, central adiposity, obesity, and chronic inflammation and further contributes to the development of atherosclerosis [5, 6]. Notably, recent researches have shown that even autoimmune thyroid disease patients in euthyroid still have more early atherosclerotic lesions [7, 8].

Obesity, like thyroid diseases, is a common disorder in the general population and it often occurs in a single individuals. A lot of studies showed that obesity is an important risk factor for many diseases, such as coronary heart disease, stroke, diabetes, and some types of cancer [9–11]. Thyroid dysfunctions, such as hypothyroidism, have been extensively investigated in obese subjects. Some studies have found that obesity increases the risk of hypothyroidism [12, 13]. Studies demonstrated that thyroid stimulating hormone (TSH) is positively correlated with body mass index (BMI), and they propose the positive rate of antibody in obese patients with subclinical hypothyroidism is also lower than that in nonobese patients [14]. While it is well known that hyperthyroidism leads to weight loss and hypothyroidism is associated with weight gain; the changes of thyroid function are discussed controversially in obesity [15]. Recent studies have shown that obesity may increase the risk of autoimmune diseases such as inflammatory bowel disease, psoriatic arthritis, and rheumatoid arthritis, suggesting a possible link between obesity and autoimmunity [16, 17]. Another study suggests that visceral adipose tissue (VAT) is a key component of the body's immune system. Immune cells affect the metabolism of adipocytes. In turn, adipocytes regulate the function of immune cells and provide energy for their activities. In addition, adipocytes themselves produce antibacterial peptides, proinflammatory cytokines, and adipokines. These substances work together to fight infections, alter the function of immune cells, and maintain metabolic balance [18]. Therefore, there may exist a possible common pathway between thyroid autoimmunity and abdominal obesity, especially with visceral adipose tissue in abdominal obesity subjects.

We hypothesize that the abdominal obesity may play a potential role in the development of autoimmune thyroid diseases. Although some observational studies have evaluated the effects of obesity on thyroid dysfunction, the relationship between abdominal obesity and autoimmune thyroid diseases remains undetermined. The data used in this study were from the Survey on Prevalence in Thyroid Diseases and Risk Factors in Xinjiang, China. The aim of the study was to define the influence of abdominal obesity on Hashimoto's thyroiditis among Xinjiang Chinese Natural population.

## 2. Materials and Methods

### 2.1. Study Participants

The study was approved by the ethics committee of the People's Hospital of Xinjiang Uyghur Autonomous Region, and all participants provided written informed consent before data collection. This is a population-based cross-sectional study and conducted in two communities of Urumqi in Xinjiang Province, during May–July, 2013. Adults aged 16 years or older who were Chinese citizens and had lived in their current residence were invited to participate in our study. The exclusion criteria comprised subjects with severe communication problems, had received thyroid therapy including medicine and/or surgery or radiotherapy and severe/acute illness, or who were unwilling to participate.

A total of 2253 subjects enrolled initially. Participants with missing laboratory results (*n* = 193), anthropometric measurement data (*n* = 137), and questionnaire data (*n* = 35) were excluded from the study. Finally, 1888 subjects were included in the final analysis (Supplementary [Supplementary-material supplementary-material-1]).

### 2.2. Data Collection

All data collections were performed by the same staff group from the Department of Endocrinology in People's Hospital of Xinjiang Uyghur Autonomous Region. All staff were trained according to a standard protocol that made them familiar with the specific tools and methods used. A standard questionnaire of each participant was administered by the trained staff group, including demographic characteristics, medical history, previous diagnoses of thyroid diseases, and whether the subject had undergone thyroid therapy, including medicine and/or surgery or radiotherapy for the head and neck. Weight, height, and waist and hip circumference were measured according to a standard protocol. All anthropometric measurements were conducted at the same time when the serum samples were collected.

### 2.3. Laboratorial Assays

Serum samples for laboratorial assays were drawn after an overnight fast of at least 8 hours. Blood samples were stored at −20°C when collected and shipped to our laboratory within 2–4 hours of collection.

Thyroid parameters, serum thyroid-stimulating hormone (TSH), and levels of thyroid peroxidase antibody (TPOAb) and thyroglobulin antibody (TgAb) were measured by a Roche electrochemiluminometric analyzer (Cobas-e601 analyzer, Roche, Mannheim, Germany). Low-density lipoprotein (LDL), high-density lipoprotein (HDL), triglycerides (TG), and total cholesterol (TC) were measured by LABOSPECT 008 AS.

### 2.4. Definition of Variables

The normal reference range for TSH is 0.27–4.2 mIU/L; for TPOAb, it is 0–35 IU/mL; and for TgAb, it is 0–116 IU/mL [19].

HT was defined as the serum TPOAb (>35 IU/mL) and/or TgAb (>116 IU/mL) positivity (TPO/TgAb (+)) (Roche).

Normal weight is defined as 18.5 ≤ BMI < 24 kg/m^2^, while overweight is defined as BMI ≥24 kg/m^2^, and obese is defined as BMI ≥28 kg/m^2^ according to the criteria [20].

Central obesity was defined as waist circumference ≥85 cm in females and ≥90 cm in males [20].

Total cholesterol <5.2 mmol/L, triglycerides <1.7 mmol/L, LDL-C <3.4 mmol/L, and HDL-C >1.0 mmol/L are considered to be the appropriate level of blood lipids according to Guidelines for the Prevention and treatment of dyslipidemia in Chinese Adults [21].

### 2.5. Statistical Analysis

We performed survey analyses with IBM SPSS Statistics, Version 20.0 (SPSS, Chicago, IL, USA). A *P* value <0.05 was considered significant. Continuous variables with normal distribution were presented as mean with standard deviation (SD), and categorical variables were presented as the median with the 25th and 75th percentiles. Continuous variables were compared using Student's *t* test. The Mann–Whitney *U* test was used for nonnormally distributed continuous variables. Correlations of TPO/TgAb positivity with metabolic and lipid parameters were analyzed using Spearman's correlation analysis. The associations among TPO/TgAb positivity and with metabolic and lipid parameters were assessed by logistic regression. Results were expressed as odds ratios (95% confidence interval). The body mass index (BMI) was calculated as weight in kilograms divided by height in meters squared.

## 3. Results

### 3.1. General Characteristics of the Study Population

A total of 1888 subjects were enrolled in this study. The mean age was 46.27 ± 14.55 (range, 16–84) years for all subjects, 46.04 ± 16.41 (range, 16–83) years in males and 46.37 ± 13.64 (range, 17–84) years in females. The mean body mass index (BMI) was 25.96 ± 4.32 kg/m^2^, waist circumference was 85.97 ± 12.11 cm, and hip circumference was 97.07 ± 8.28 cm. Among them, 585 (31%) were males and 1303 (69%) were females. There were evident differences in TSH, TPOAb, TgAb, HDL, TG, waist circumference, and hip circumference levels between men and women (*P* < 0.01).

Men had worse lipid profile compared with women: they had significantly lower HDL cholesterol levels, 1.25 ± 0.27 versus 1.48 ± 0.48 mmol/L (*P* < 0.01), and higher TG levels, 1.54 (0.99–2.14) versus 1.16 (0.85–1.64) mmol/L (*P* < 0.01). There was no significant difference in TC and LDL cholesterol levels between the two gender groups (*P* > 0.05).

Thyroid parameters between subjects in men and women also varied significantly. Women subjects showed higher TSH levels (2.66 (1.68–4.10) versus 2.06 (1.45–3.16) uIU/ml, *P* < 0.01), TPOAb (27.77 (22.61–38.48) versus 24.57 (19.60–31.72) IU/ml, *P* < 0.01), and TgAb (31.13 (26.33–93.67) versus 27.53 (24.50–32.05) IU/ml, *P* < 0.01).

When metabolic parameters were evaluated, men had higher waist circumference (90.24 ± 11.84 versus 84.06 ± 11.73 cm, *P* < 0.01) and hip circumference levels (97.83 ± 7.57 versus 96.72 ± 8.55 cm, *P* < 0.01) than women subjects. However, there were no significant differences in BMI between the two gender groups (26.14 ± 3.75 versus 25.88 ± 4.55 kg/m^2^, *P* > 0.05).

Characteristics of the study population, their anthropometric data, metabolic parameters, and thyroid parameters are shown in Supplementary [Supplementary-material supplementary-material-1].

### 3.2. Clinical Characteristics according to TPOAb and TgAb Levels

The prevalence of TPOAb and/or TgAb positive (TPO/TgAb (+)) was 32.1% (21.2% in men and 37% in women, *P* < 0.01; Supplementary [Supplementary-material supplementary-material-1]). The prevalence of overweight and obesity was higher in men (71.1%) than women (63.5%) in this population (*P* < 0.01; Supplementary [Supplementary-material supplementary-material-1]). Men had a higher prevalence of abdominal obesity than women (56.6% in men and 47.6% in women, *P* < 0.01) (Supplementary [Supplementary-material supplementary-material-1]).

The characteristics of the participants in terms of TPO/TgAb positivity are summarized in Supplementary [Supplementary-material supplementary-material-1].

For men, participants with HT had a significantly higher level of waist circumference compared with participants in the TPOAb and TgAb-negative (TPO and TgAb (−)) group (*P* < 0.05). BMI was slightly higher in the HT group but did not reach statistical significance (26.51 ± 4.12 versus 26.04 ± 3.64 kg/m^2^, *P* > 0.05). There was no significant difference in hip circumference, TC, HDL, and LDL cholesterol levels between the two groups (*P* > 0.05).

For women, no significant difference was found between the HT (+) and HT (−) groups with regard to BMI, waist circumference, hip circumference, TC, HDL, and LDL cholesterol levels (*P* > 0.05).

### 3.3. Association of TPO/TgAb Positivity with Metabolic and Lipid Parameters

The Spearman's correlation analysis showed a correlation between TPO/TgAb positivity with metabolic and lipid parameters. It was found that TPOAb correlates positively with waist circumference (*r* = 0.100, *P* < 0.05), TC (*r* = 0.051, *P* < 0.05) in men, while TPOAb correlates positively with hip circumference (*r* = 0.084, *P* < 0.01) and TC (*r* = 0.081, *P* < 0.05) in women (Supplementary [Supplementary-material supplementary-material-1]). Given that the findings of TPO positivity were positively associated with metabolic parameters, we evaluated the adjusted odds ratios (ORs) for metabolic in the TPOAb (+) group. Adjusted ORs were calculated after adjusting for age and TSH using the binary logistic regression model. As shown in Supplementary [Supplementary-material supplementary-material-1], TPOAb positivity was associated with an increased risk of abdominal obesity in men, and the OR was 1.104 (95% CI 1.035, 1.151, *P* < 0.05). General obesity and hyperlipidaemia (hypercholesterolemia, hypertriglyceridemia, hyper-low-density lipoproteinemia) had no significant relationship with TPOAb positivity in men subjects (*P* < 0.05). No significant association of TPOAb/TgAb positivity with obesity, abdominal obesity, and hyperlipidaemia was found in women subjects (*P* < 0.05).

## 4. Discussion

In our study, the prevalence of HT was 32.1% (TPOAb positivity 25.7%, TgAb positivity 18.6%). Compared with the previous epidemiological investigation of thyroid diseases conducted by Teng et al. [22] in 10 cities in China (the prevalence positive TPOAb 11.5%, positive TgAb 12.6%), this study showed that the positive rate of thyroid autoantibodies in Xinjiang was higher. Our study showed that nearly two-thirds of participants are either overweight (37%) or obese (28.9%), and more than half of the participants are abdominal obese (50.4%) and are higher than the data from the 2009 China Health and Nutrition Survey (obese 26.4%; abdominal obese 37.4%) [23].

The susceptibility to autoimmune thyroid disease depends mainly on the genetic determinants of HLA and non-HLA loci (CTLA4, CD40, PTPN22, TG, and TSH-R genes), and these genetic determinants may be affected by various environmental factors, such as iodine deficiency, stress, drugs, chemical contaminants, and infectious organisms [24]. The prevalence of HT is 21.2% in men and 37% in women in our study. Consistent with previous researches [3], the positive thyroid autoantibodies showed substantial correlation with female gender in this research. TPOAb was identified as a major autoantigen, and the presence of TPOAb might be correlated with severity of thyroid lymphocytic infiltration, despite the presence or absence of hypothyroidism [25]. Although genetic and environmental factors are known contributors of AITD, a causal relationship between obesity and thyroid autoimmunity has not been established, but observational data from the general population suggest that obesity may increase the risk of autoimmune diseases, such as rheumatoid arthritis and psoriatic arthritis [26, 27], probably due to the chronic proinflammatory state caused by the accumulation of VAT in obese patients. Compared with Caucasians, Chinese people are less obese and body fat distribution tends to accumulate in the abdominal cavity, which is more likely to form abdominal obesity. The overweight and obesity were significantly higher among male (41.2% and 29.9%) than among female (35.1% and 28.4%) participants in this study. Moreover, men had a higher prevalence of abdominal obesity than women (56.6% in men and 47.6% in women). The prevalence of overweight, general obesity, and abdominal obesity in our study is higher than the data from China Health and Nutrition Survey (CHNS) [28]. However, the same as CHNS, the prevalence of overweight and general obesity is higher in men than women in our study. Meanwhile, there has been a sharp increase in the prevalence of abdominal obesity in men than in women in this study. Explanations for the higher prevalence of abdominal obesity in men than that in women might be due to the increased intake of beer by men and the different lifestyle, such as increased intake of high-fat diet and less exercise. Other explanations for the greater increase of abdominal obesity prevalence among men than women might be sex hormone responses to obesogenic environmental changes [29].

We also found worse lipid levels, such as had higher serum TG levels and lower HDL levels in men than in women in our study. Our data also showed that TPOAb titers was positively associated with TC, both in men and women. A similar finding was concluded that TPOAb-positive causes dyslipidemia in normal thyroid populations [30], while another study recorded no significant differences between TPOAb-positive and TPOAb-negative groups [31].

Results of the current study revealed that men with TPOAb (+) and/or TgAb (+) have a higher waist circumference than women. Notably, the waist circumference was positively associated with TPOAb in men but not in women, while hip circumference was positively associated with TPOAb in women but not in men. Our present findings also suggested that abdominal obesity is a risk factor for TPOAb positivity in men. Therefore, we consider that there is a gender difference in the relationship between obesity and HT in Chinese adults. The gender difference in the effects of sex hormones on HT between men and women may be due to the different roles of estradiol and testosterone in regulating immune responses [32, 33]. Another possible explanation is that the gender differences in the associations of HT and obesity may be related to gender differences in the body fat distribution between men and women, which may lead to gender-specific changes in peripheral metabolism of thyroid hormones [34, 35]. Obesity can lead to different and abnormal changes in sex hormones in men and women, which can lead to specific gender risks in thyroid autoimmune diseases. The gender association of obesity with thyroid autoimmune diseases may result from differences in adipokines between men and women [36]. Many adipokines, such as leptin and adiponectin, play an important role in regulating immunity and are considered to be a key link between obesity and obesity-related diseases. Therefore, the sex-specific effects of adipokines may thus be involved in the gender specificity of obesity with thyroid autoimmune diseases [37]. In obesity, it can directly or indirectly affect immune tolerance by altering the secretion of adipokines (mainly leptin, adiponectin, and mucin) and/or cytokines (interleukin-6, tumor necrosis factor alpha, and interleukin) [37]. The end result will be a shift from a Th2 to Th1 immune response, which is more likely to produce an autoimmune response. It is well established that *T* helper cell 1 (Th1) type cytokines play pivotal roles in Hashimoto's thyroiditis and Graves' disease. Concordantly, cytokines that counteract Th1 responses, produced by Th2 cell subsets and *T* regulatory cells, has been shown to have a protective effect on autoimmunity [38]. Our previous studies have found that an increased immune deviation of Th1 lymphocytes and compensatory accelerating activity of Treg cells in HT patients [39]. The visceral adipose tissue (VAT) includes macrophages, endothelial cells, and T cells with biased T cell receptors that may promote an immune response by producing an excess of proinflammatory cytokines [40]. In addition, VAT is a reservoir of regulatory T cells (Treg), and a small subset (5–15%) of the T cell compartment is capable of controlling autoimmune responses. Researches showed that Treg cells are affected by leptin and involved in the control of autoimmunity [41] and thyroid cell apoptosis [42]. Another study suggests that leptin regulates the immune response of Thl [43]. The adipocytokines leptin and IL-6 inhibit regulatory T cells [26], whereas obesity alters cell-mediated Th1 immune responses, resulting in CD3 and CD4 T helper cells and CD8 T suppressor/cytotoxic cells defects [44, 45]. Some researchers have pointed out that obesity increases the risk of thyroid autoimmunity, which is related to leptin levels and other known predictors [13]. However, until now, the underlying mechanism of the association between abdominal obesity, especially visceral fat, and thyroid autoimmune diseases has not been clear.

Our results confirmed a relationship between obesity and AITD, especially abdominal obesity in men. Although women have a higher incidence of autoimmune diseases including AITD, the results of our research indicate that abdominal obesity is also a risk factor for TPOAb positivity in men. In terms of different ethnicities, race, and age, gender composition may have accounted for the difference in results obtained.

Our study has several strengths. First, only a few previous studies about the association of obesity, especially central obesity with thyroid autoimmunity in a large Chinese population, were available. We evaluated a relatively large sample of participants to examine the association of TPOAb and TgAb with obesity. Second, the data are highly reliable because all anthropometry and questionnaires are performed by the same trained research group with strict quality control, and all tests are performed under the same equipment and protocols. Third, we recruited community residents in Xinjiang China to make the results more representative. However, this study also has several limitations. First, because of the cross-sectional study, the direction of any causal relationship cannot be determined and the observed effect need to continue to verify in other people; thus, the prospective studies are needed. This study is the first part of our series of researches about the possible relationship between autoimmune thyroid disease and visceral adipose tissue and possible pathogenesis. Our subsequent follow-up study may make up for this limitation. Second, there is lack of a complete definition of chronic autoimmune thyroiditis. Third, other social and environmental variables, such as work stress, dietary habits, and sedentary lifestyles, which would have impact on obesity and thyroid autoimmunity, were not considered.

In conclusion, our present evaluation of the association of autoimmune thyroid disease with obesity, especially abdominal obesity, and dyslipidemia, has revealed that the prevalence of overweight, obesity, and abdominal obesity was higher in men. The waist circumference of men with Hashimoto's thyroiditis was higher than that of HT (−). The current study also showed that abdominal obesity can enhance the risk of thyroid autoimmunity. Further researches about the relationship between the abdominal obesity and thyroid diseases are warranted. Therefore, we suggest a follow-up study to understand the potential mechanism of visceral adipose tissue may contribute to promote susceptibility to thyroid autoimmunity.

## Data Availability

The data used to support the findings of this study are available from the corresponding author upon request.

## Supplementary Materials

Supplementary Materials Supplementary Table 1: clinical characteristics of the study participants. Supplementary Table 2: prevalence of Hashimoto's thyroiditis and obesity. Supplementary Table 3: characteristics of subjects in terms of the level of serum TPOAb and TgAb. Supplementary Table 4: correlation between metabolic and lipid parameters with serum TPOAb and TgAb levels. Supplementary Figure 1: flow diagram of participant enrollment in our study. Supplementary Figure 2: associations of TPOAb positivity with obesity, abdominal obesity, and hyperlipidaemia in men.
