# Supplementary material for: Impact of Abdominal Obesity on Thyroid Auto-Antibody Positivity: Abdominal Obesity Can Enhance the Risk of Thyroid Autoimmunity in Men
Source: Int J Endocrinol. 2020 Mar 13;2020:6816198. doi: 10.1155/2020/6816198 (PMC7093900; doi:10.1155/2020/6816198)

Table 1. Clinical characteristics of the study participants.

Characteristics	Total	Male	Female	P value
N (%)	1888	585（31%）	1303（69%）	-
Age (year)	46.27±14.55	46.04±16.41	46.37±13.64	0.430
BMI (kg/m2)	25.96±4.32	26.14±3.75	25.88±4.55	0.282
WC (cm)	85.97±12.11	90.24±11.84	84.06±11.73	0.0001
HC (cm)	97.07±8.28	97.83±7.57	96.72±8.55	0.005
TSH (mIU/L)	2.42（1.59-3.80）	2.06（1.45-3.16）	2.66（1.68-4.10）	0.0001
TPOAb（IU/mL）	26.89（21.62-35.51）	24.57（19.60-31.72）	27.77（22.61-38.48）	0.0001
TgAb（IU/mL）	29.80（25.60-48.01）	27.53（24.50-32.05）	31.13（26.33-93.67）	0.0001
TC (mmol/L)	4.69±1.05	4.65±1.10	4.71±1.02	0.164
TG (mmol/L)	1.25（0.89-1.80）	1.54（0.99-2.14）	1.16（0.85-1.64）	0.0001
HDL-C (mmol/L)	1.41±0.44	1.25±0.27	1.48±0.48	0.0001
LDL-C (mmol/L)	2.71±0.78	2.76±0.79	2.69±0.78	0.104

BMI, Body mass index; WC, Waist circumference; HC: Hip circumference；TSH, Thyroid-stimulating hormone; TPOAb, thyroid peroxidase antibody; TgAb, Thyroglobulin antibody; LDL-C, Low-density lipoprotein cholesterol; HDL-C, High-density lipoprotein cholesterol; TG, Triglycerides; TC, Total cholesterol.

Table 2. Prevalence of Hashimoto's thyroiditis and Obesity.

	Hashimoto's thyroiditis	Overweight and Obesity			Abdominal obesity
		Total	Overweight	Obesity	
Total, N (%)	606 (32.1%)	1244 (65.9%)	699 (37%)	545 (28.9%)	951 (50.4%)
Male, N (%)	124 (21.2%)	416 (71.1%)	241 (41.2%）	175 (29.9%）	331 (56.6%)
Female,N (%)	482 (37%)	828 (63.5%)	458 (35.1%）	370 (28.4%）	620 (47.6%)
X^2	46.215	10.282	6.332	0.453	13.078
P value	0.0001	0.001	0.012	0.501	0.0001

Table 3. Characteristics of subjects in terms of the level of serum TPOAb and TgAb.

	TPO and/or TgAb (+)	TPO and TgAb (−)	P value
Male			
N (%)	124 (21.2%)	461 (78.8%)	-
Age (year)	46.06±15.23	45.08±16.60	0.306
BMI (kg/m2)	26.51±4.12	26.04±3.64	0.211
WC (cm)	92.10±12.66	89.74±11.57	0.049
HC (cm)	98.89±9.12	97.54±7.09	0.130
TSH (mIU/L)	2.80 (1.59-4.01)	1.98 (1.43-2.94）	0.0001
TC (mmol/L)	4.76±1.02	4.62±1.12	0.232
TG (mmol/L)	1.6 (1.01-2.22)	1.54 (0.96-2.13)	0.316
HDL (mmol/L)	1.24±0.22	1.25±0.28	0.543
LDL (mmol/L)	2.75±0.75	2.76±0.80	0.886
Female			
N (%)	482 (37%)	821 (63%)	-
Age (year)	46.05±12.92	46.05±14.05	0.264
BMI (kg/m2)	26.01±4.84	25.81±4.36	0.454
WC (cm)	84.16±12.20	84.0±11.45	0.990
HC (cm)	96.72±8.89	96.73±8.35	0.809
TSH (mIU/L)	3.0 (1.71-4.93)	2.48 (1.67-3.76)	0.0001
TC (mmol/L)	4.73±1.03	4.69±1.02	0.567
TG (mmol/L)	1.13 (0.84-1.64)	1.18 (0.85-1.64)	0.867
HDL (mmol/L)	1.50±0.65	1.47±0.33	0.324
LDL (mmol/L)	2.66±0.78	2.70±0.78	0.468

BMI, Body mass index; WC, Waist circumference; HC: Hip circumference；TSH, Thyroid-stimulating hormone; TPOAb, thyroid peroxidase antibody; TgAb, Thyroglobulin antibody; LDL-C, Low-density lipoprotein cholesterol; HDL-C, High-density lipoprotein cholesterol; TG, Triglycerides; TC, Total cholesterol.

Table 4 Correlation between metabolic and lipid parameters with serum TPOAb, TgAb levels.

	TPOAb				TgAb			
	Male		Female		Male		Female	
	γ	***p***	γ	***p***	γ	***p***	γ	***p***
BMI	0.065	0.116	0.041	0.137	0.029	0.483	0.015	0.583
WC	0.100	0.016	0.030	0.284	0.052	0.210	-0.002	0.945
HC	0.090	0.130	0.084	0.001	0.029	0.479	0.013	0.642
TC	0.051	0.026	0.081	0.019	0.000	0.992	0.003	0.915
TG	0.060	0.149	0.012	0.666	0.030	0.472	0.006	0.840
HDL	0.059	0.151	0.023	0.416	-0.032	0.444	0.010	0.720
LDL	0.010	0.816	0.004	0.891	0.000	0.991	0.008	0.766

BMI, Body mass index; WC, Waist circumference; HC: Hip circumference；TSH, Thyroid-stimulating hormone; TPOAb, thyroid peroxidase antibody; TgAb, Thyroglobulin antibody; LDL-C, Low-density lipoprotein cholesterol; HDL-C, High-density lipoprotein cholesterol; TG, Triglycerides; TC, Total cholesterol.

Figure 1. Flow diagram of participant enrollment in our study.

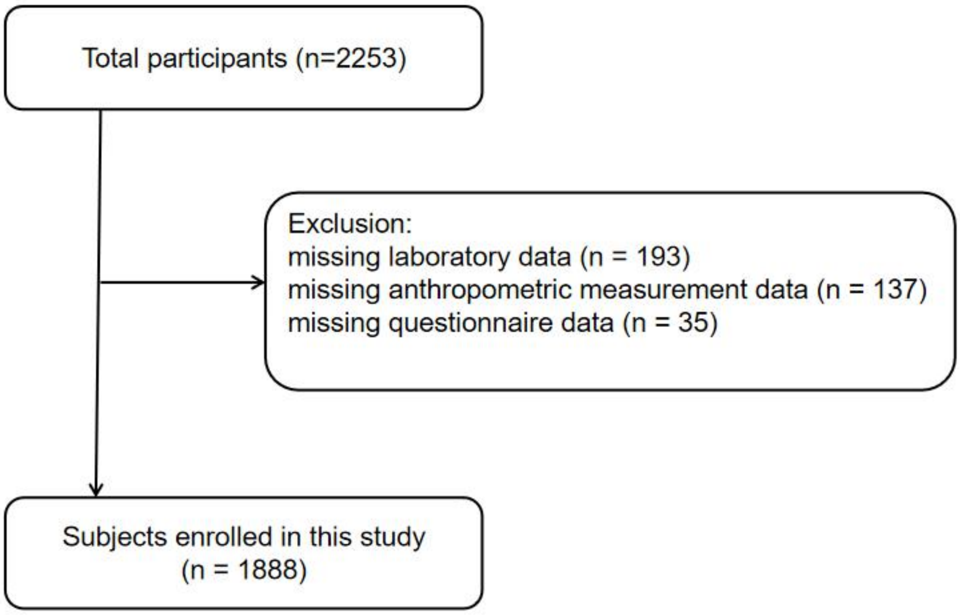

Figure 2. Associations of TPOAb positivity with obesity, abdominal obesity and hyperlipidemia in men.

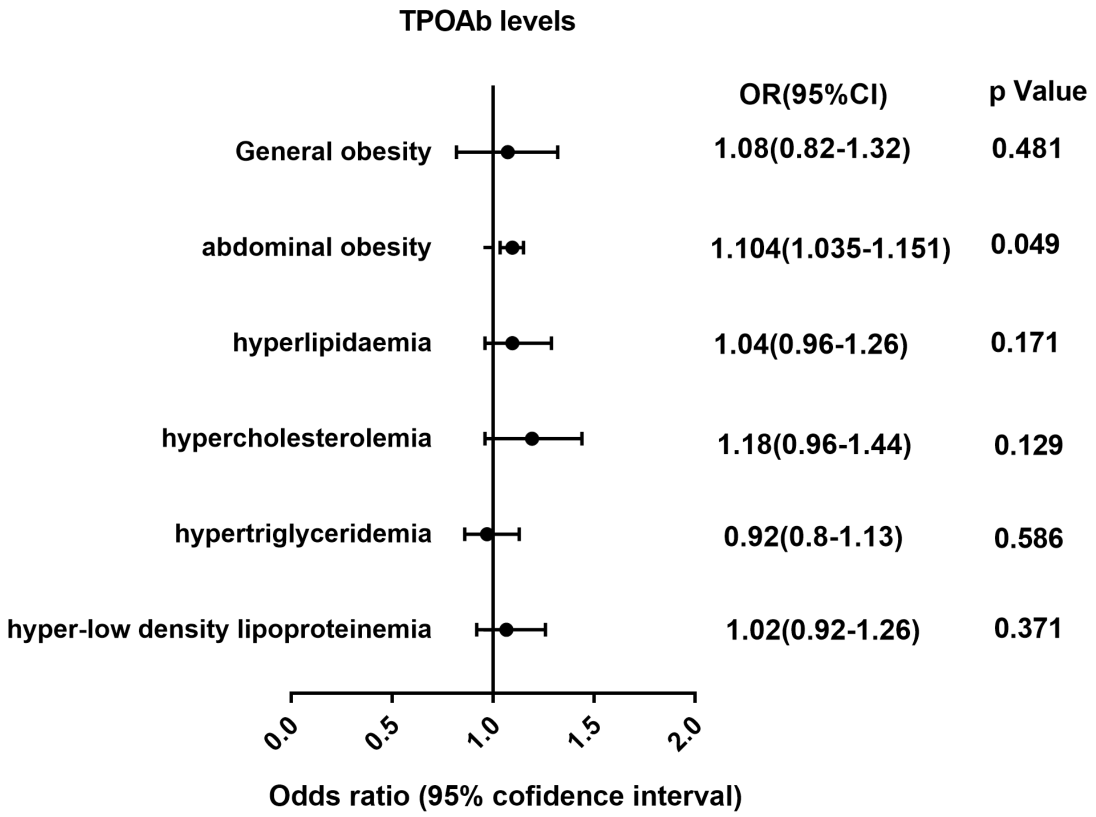

Supplement: Supplementary Materials — Supplementary Table 1: clinical characteristics of the study participants. Supplementary Table 2: prevalence of Hashimoto's thyroiditis and obesity. Supplementary Table 3: characteristics of subjects in terms of the level of serum TPOAb and TgAb. Supplementary Table 4: correlation between metabolic and lipid parameters with serum TPOAb and TgAb levels. Supplementary Figure 1: flow diagram of participant enrollment in our study. Supplementary Figure 2: associations of TPOAb positivity with obesity, abdominal obesity, and hyperlipidaemia in men. [file 6816198.f1.zip › Supplementary materials.pdf]
