# Supplementary figures and images for: Impact of Abdominal Obesity on Thyroid Auto-Antibody Positivity: Abdominal Obesity Can Enhance the Risk of Thyroid Autoimmunity in Men
Source: Int J Endocrinol. 2020 Mar 13;2020:6816198. doi: 10.1155/2020/6816198 (PMC7093900; doi:10.1155/2020/6816198)

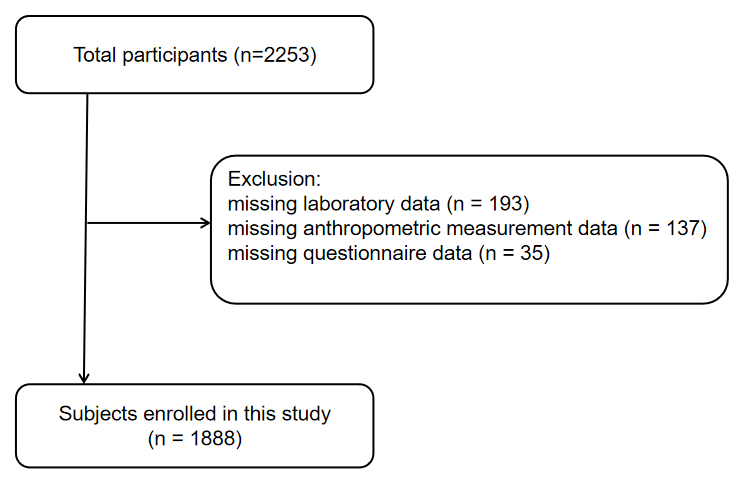

Supplement: Supplementary Materials — Supplementary Table 1: clinical characteristics of the study participants. Supplementary Table 2: prevalence of Hashimoto's thyroiditis and obesity. Supplementary Table 3: characteristics of subjects in terms of the level of serum TPOAb and TgAb. Supplementary Table 4: correlation between metabolic and lipid parameters with serum TPOAb and TgAb levels. Supplementary Figure 1: flow diagram of participant enrollment in our study. Supplementary Figure 2: associations of TPOAb positivity with obesity, abdominal obesity, and hyperlipidaemia in men. [file 6816198.f1.zip › Figure 1.png]

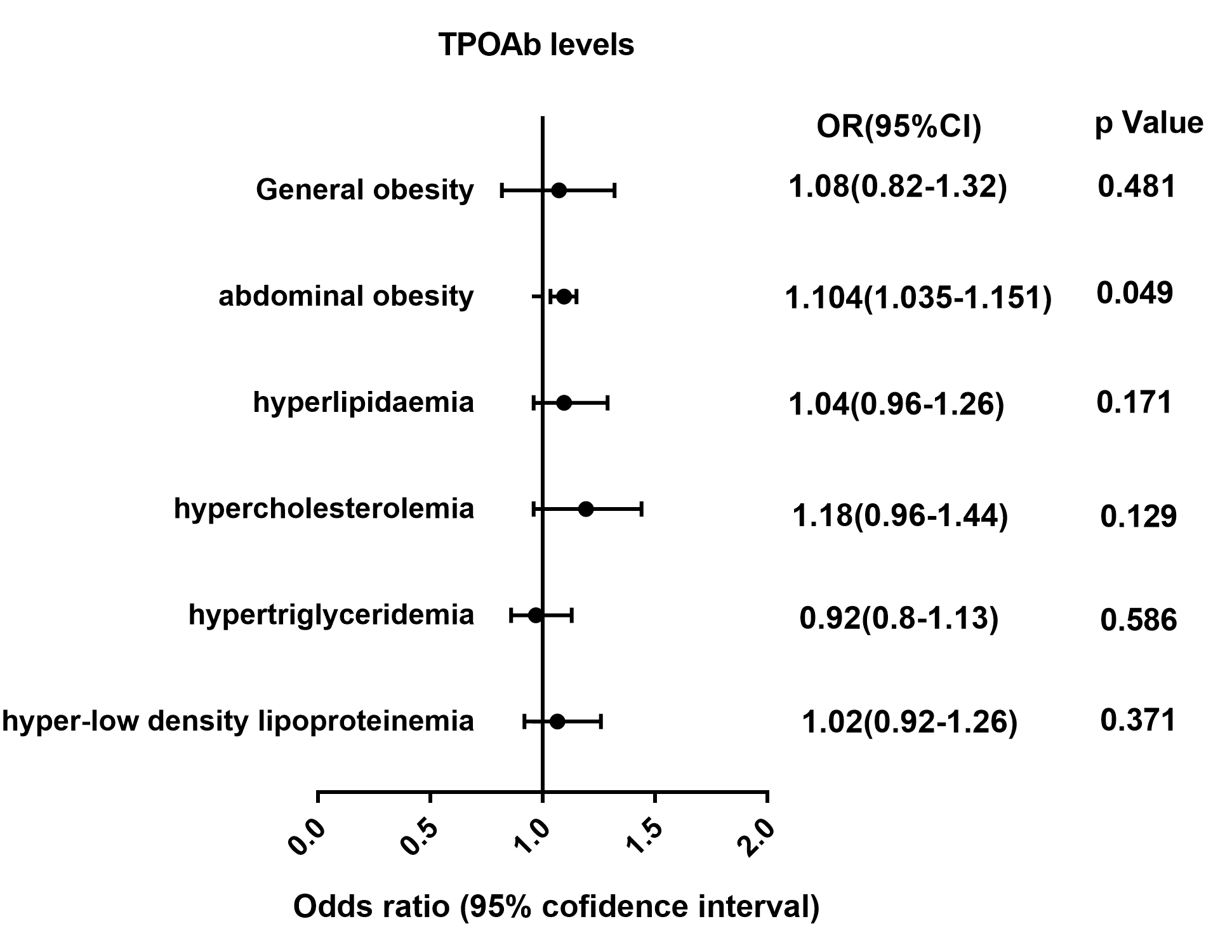

Supplement: Supplementary Materials — Supplementary Table 1: clinical characteristics of the study participants. Supplementary Table 2: prevalence of Hashimoto's thyroiditis and obesity. Supplementary Table 3: characteristics of subjects in terms of the level of serum TPOAb and TgAb. Supplementary Table 4: correlation between metabolic and lipid parameters with serum TPOAb and TgAb levels. Supplementary Figure 1: flow diagram of participant enrollment in our study. Supplementary Figure 2: associations of TPOAb positivity with obesity, abdominal obesity, and hyperlipidaemia in men. [file 6816198.f1.zip › Figure 2.tif]
