# Supplementary material for: Impact of Abdominal Obesity on Thyroid Auto-Antibody Positivity: Abdominal Obesity Can Enhance the Risk of Thyroid Autoimmunity in Men
Source: Int J Endocrinol. 2020 Mar 13;2020:6816198. doi: 10.1155/2020/6816198 (PMC7093900; doi:10.1155/2020/6816198)
Supplement: Supplementary Materials — Supplementary Table 1: clinical characteristics of the study participants. Supplementary Table 2: prevalence of Hashimoto's thyroiditis and obesity. Supplementary Table 3: characteristics of subjects in terms of the level of serum TPOAb and TgAb. Supplementary Table 4: correlation between metabolic and lipid parameters with serum TPOAb and TgAb levels. Supplementary Figure 1: flow diagram of participant enrollment in our study. Supplementary Figure 2: associations of TPOAb positivity with obesity, abdominal obesity, and hyperlipidaemia in men. [file 6816198.f1.zip › Supplementary materials.doc]

Table 1. Clinical characteristics of the study participants.

| Characteristics | Total | Male | Female | P value |
| --- | --- | --- | --- | --- |
| N (%) | 1888 | 585（31%） | 1303（69%） | - |
| Age (year) | 46.27±14.55 | 46.04±16.41 | 46.37±13.64 | 0.430 |
| BMI (kg/m2) | 25.96±4.32 | 26.14±3.75 | 25.88±4.55 | 0.282 |
| WC (cm) | 85.97±12.11 | 90.24±11.84 | 84.06±11.73 | 0.0001 |
| HC (cm) | 97.07±8.28 | 97.83±7.57 | 96.72±8.55 | 0.005 |
| TSH (mIU/L) | 2.42（1.59-3.80） | 2.06（1.45-3.16） | 2.66（1.68-4.10） | 0.0001 |
| TPOAb（IU/mL） | 26.89（21.62-35.51） | 24.57（19.60-31.72） | 27.77（22.61-38.48） | 0.0001 |
| TgAb（IU/mL） | 29.80（25.60-48.01） | 27.53（24.50-32.05） | 31.13（26.33-93.67） | 0.0001 |
| TC (mmol/L) | 4.69±1.05 | 4.65±1.10 | 4.71±1.02 | 0.164 |
| TG (mmol/L) | 1.25（0.89-1.80） | 1.54（0.99-2.14） | 1.16（0.85-1.64） | 0.0001 |
| HDL-C (mmol/L) | 1.41±0.44 | 1.25±0.27 | 1.48±0.48 | 0.0001 |
| LDL-C (mmol/L) | 2.71±0.78 | 2.76±0.79 | 2.69±0.78 | 0.104 |

BMI, Body mass index; WC, Waist circumference; HC: Hip circumference；TSH, Thyroid-stimulating hormone; TPOAb, thyroid peroxidase antibody; TgAb, Thyroglobulin antibody; LDL-C, Low-density lipoprotein cholesterol; HDL-C, High-density lipoprotein cholesterol; TG, Triglycerides; TC, Total cholesterol.

Table 2. Prevalence of Hashimoto's thyroiditis and Obesity.

|  | Hashimoto's thyroiditis | Overweight and Obesity | | | Abdominal obesity |
| --- | --- | --- | --- | --- | --- |
|  |  | Total | Overweight | Obesity |  |
| Total, N (%) | 606 (32.1%) | 1244 (65.9%) | 699 (37%) | 545 (28.9%) | 951 (50.4%) |
| Male, N (%) | 124 (21.2%) | 416 (71.1%) | 241 (41.2%） | 175 (29.9%） | 331 (56.6%) |
| Female,N (%) | 482 (37%) | 828 (63.5%) | 458 (35.1%） | 370 (28.4%） | 620 (47.6%) |
| X^2^ | 46.215 | 10.282 | 6.332 | 0.453 | 13.078 |
| P value | 0.0001 | 0.001 | 0.012 | 0.501 | 0.0001 |

Table 3. Characteristics of subjects in terms of the level of serum TPOAb and TgAb.

|  | TPO and/or TgAb (+) | TPO and TgAb (−) | P value |
| --- | --- | --- | --- |
| Male | | | |
| N (%) | 124 (21.2%) | 461 (78.8%) | - |
| Age (year) | 46.06±15.23 | 45.08±16.60 | 0.306 |
| BMI (kg/m2) | 26.51±4.12 | 26.04±3.64 | 0.211 |
| WC (cm) | 92.10±12.66 | 89.74±11.57 | 0.049 |
| HC (cm) | 98.89±9.12 | 97.54±7.09 | 0.130 |
| TSH (mIU/L) | 2.80 (1.59-4.01) | 1.98 (1.43-2.94） | 0.0001 |
| TC (mmol/L) | 4.76±1.02 | 4.62±1.12 | 0.232 |
| TG (mmol/L) | 1.6 (1.01-2.22) | 1.54 (0.96-2.13) | 0.316 |
| HDL (mmol/L) | 1.24±0.22 | 1.25±0.28 | 0.543 |
| LDL (mmol/L) | 2.75±0.75 | 2.76±0.80 | 0.886 |
| Female | | | |
| N (%) | 482 (37%) | 821 (63%) | - |
| Age (year) | 46.05±12.92 | 46.05±14.05 | 0.264 |
| BMI (kg/m2) | 26.01±4.84 | 25.81±4.36 | 0.454 |
| WC (cm) | 84.16±12.20 | 84.0±11.45 | 0.990 |
| HC (cm) | 96.72±8.89 | 96.73±8.35 | 0.809 |
| TSH (mIU/L) | 3.0 (1.71-4.93) | 2.48 (1.67-3.76) | 0.0001 |
| TC (mmol/L) | 4.73±1.03 | 4.69±1.02 | 0.567 |
| TG (mmol/L) | 1.13 (0.84-1.64) | 1.18 (0.85-1.64) | 0.867 |
| HDL (mmol/L) | 1.50±0.65 | 1.47±0.33 | 0.324 |
| LDL (mmol/L) | 2.66±0.78 | 2.70±0.78 | 0.468 |

BMI, Body mass index; WC, Waist circumference; HC: Hip circumference；TSH, Thyroid-stimulating hormone; TPOAb, thyroid peroxidase antibody; TgAb, Thyroglobulin antibody; LDL-C, Low-density lipoprotein cholesterol; HDL-C, High-density lipoprotein cholesterol; TG, Triglycerides; TC, Total cholesterol.

Table 4 Correlation between metabolic and lipid parameters with serum TPOAb, TgAb levels.

|  | TPOAb | | | | TgAb | | | |
| --- | --- | --- | --- | --- | --- | --- | --- | --- |
|  | Male | | Female | | Male | | Female | |
|  | **γ** | ***p*** | **γ** | ***p*** | **γ** | ***p*** | **γ** | ***p*** |
| BMI | 0.065 | 0.116 | 0.041 | 0.137 | 0.029 | 0.483 | 0.015 | 0.583 |
| WC | 0.100 | 0.016 | 0.030 | 0.284 | 0.052 | 0.210 | -0.002 | 0.945 |
| HC | 0.090 | 0.130 | 0.084 | 0.001 | 0.029 | 0.479 | 0.013 | 0.642 |
| TC | 0.051 | 0.026 | 0.081 | 0.019 | 0.000 | 0.992 | 0.003 | 0.915 |
| TG | 0.060 | 0.149 | 0.012 | 0.666 | 0.030 | 0.472 | 0.006 | 0.840 |
| HDL | 0.059 | 0.151 | 0.023 | 0.416 | -0.032 | 0.444 | 0.010 | 0.720 |
| LDL | 0.010 | 0.816 | 0.004 | 0.891 | 0.000 | 0.991 | 0.008 | 0.766 |

BMI, Body mass index; WC, Waist circumference; HC: Hip circumference；TSH, Thyroid-stimulating hormone; TPOAb, thyroid peroxidase antibody; TgAb, Thyroglobulin antibody; LDL-C, Low-density lipoprotein cholesterol; HDL-C, High-density lipoprotein cholesterol; TG, Triglycerides; TC, Total cholesterol.

Figure 1. Flow diagram of participant enrollment in our study.

Figure 2. Associations of TPOAb positivity with obesity, abdominal obesity and hyperlipidemia in men.
